# A Clinical Assessment of an Ultrasound Computer-Aided Diagnosis System in Differentiating Thyroid Nodules With Radiologists of Different Diagnostic Experience

**DOI:** 10.3389/fonc.2020.557169

**Published:** 2020-09-11

**Authors:** Yichun Zhang, Qiong Wu, Yutong Chen, Yan Wang

**Affiliations:** ^1^Department of Ultrasound in Medicine, Shanghai Sixth People’s Hospital Affiliated to Shanghai Jiao Tong University, Shanghai, China; ^2^Shanghai Institute of Ultrasound in Medicine, Shanghai, China

**Keywords:** thyroid nodule, ultrasonography, computer-aided system, diagnosis, thyroid cancer

## Abstract

**Introduction:**

This study aimed to assess the diagnostic performance and the added value to radiologists of different levels of a computer-aided diagnosis (CAD) system for the detection of thyroid cancers.

**Methods:**

303 patients who underwent thyroidectomy from October 2018 to July 2019 were retrospectively reviewed. The diagnostic performance of the senior radiologist, the junior radiologist, and the CAD system were compared. The added value of the CAD system was assessed and subgroup analyses were performed according to the size of thyroid nodules.

**Results:**

In total, 186 malignant thyroid nodules, and 179 benign thyroid nodules were included; 168 were papillary thyroid carcinoma (PTC), 7 were medullary thyroid carcinoma (MTC), 11 were follicular carcinoma (FTC), 127 were follicular adenoma (FA) and 52 were nodular goiters. The CAD system showed a comparable specificity as the senior radiologist (86.0% vs. 86.0%, *p* > 0.99), but a lower sensitivity and a lower area under the receiver operating characteristic (AUROC) curve (sensitivity: 71.5% vs. 95.2%, *p* < 0.001; AUROC: 0.788 vs. 0.906, *p* < 0.001). The CAD system improved the diagnostic sensitivities of both the senior and the junior radiologists (97.8% vs. 95.2%, *p* = 0.063; 88.2% vs. 75.3%, *p* < 0.001).

**Conclusion:**

The use of the CAD system using artificial intelligence is a potential tool to distinguish malignant thyroid nodules and is preferable to serve as a second opinion for less experienced radiologists to improve their diagnosis performance.

## Introduction

The incidence of thyroid nodules, up to 68% of the general population, continues to show increasing growth (1, 2). As one of the most extensively applied methods in the detection of thyroid nodules, the ultrasound has the advantages of accessibility, cost-effectiveness, and non-radiation. Although the particular ultrasound (US) features such as microcalcifications, hypoechogenicity, and irregular margins are commonly considered to relate to malignant thyroid disease, the presence of interobserver variation is inevitable (3, 4). Compared with seasoned radiologists, less experienced radiologists are at a greater risk of a misleading diagnosis of thyroid cancer.

Computer-aided diagnosis (CAD) has attracted great attention of researchers as a newly developed technique that has potential in enhancing radiologists’ interpretation and overcoming subjective limitations. The CAD detection and diagnosis methods are based on machine learning approaches that extract features based on shape, texture, and statistical values, differentiating benign and malignant nodules (5–7).

Several studies have shown that CAD system has comparable performance to radiologists in terms of sensitivity (8–10). However, few studies have compared the distinction of diagnosis performance between the CAD system and radiologists with various levels of experience in the diagnosis of thyroid cancer and no detailed study has been conducted to focus on the influence of nodule size on CAD performance. Therefore, this retrospective study aimed to validate the clinical role of the CAD systems in thyroid cancer diagnosis and to evaluate their future developmental directions.

## Materials and Methods

### Ethics and Consent

This prospective study was approved by our Institutional Review Board, and the requirement for informed consent was waived due to its retrospective nature.

### Database

We retrospectively reviewed medical records of 303 patients who were treated at our center from October 2018 to July 2019. Patients who received an ultrasound examination prior to scheduled surgery with sufficient clinical information were enrolled. The histopathologic diagnosis of the nodules was established by surgery. Finally, there were 186 malignant nodules and 179 benign nodules included in our study.

### Ultrasound Images Acquisition and Radiology Analysis

The US scans were operated with a 12–18 MHz linear probe (ACUSON S2000; Siemens Medical Solutions, Mountain View, CA, United States). The US images presented in a random fashion were assessed by a senior radiologist of more than 10 years’ experience and a junior radiologist of 2 years’ experience.

**FIGURE 1:**
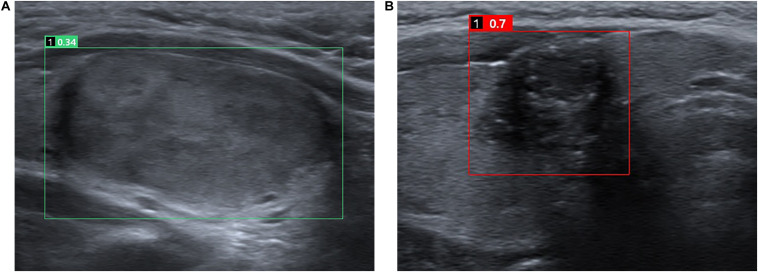
Representative cases of benign **(A)** and malignant **(B)** thyroid nodules. For the benign nodule **(A)**, both the CAD system and the senior and the junior radiologists diagnosed it as a benign nodule. The CAD system rate the nodule of 0.34. For the malignant nodule **(B)**, both the CAD system and the senior and the junior radiologists diagnosed it as a malignant nodule. The CAD system rate the nodule of 0.7.

The CAD system used in the study was AI-SONIC for thyroid nodule (AI-SONIC; Demestics Medical Technology Co., Zhejiang, China), which can analyze the US images for real-time. A grayscale image of a transverse plane of each nodule was uploaded to the software and analyzed. The software is able to automatically mark the suspicious lesion with a square and rate the nodule on a scale of zero to one, with higher scores indicating the higher the risk of malignancy (Figure 1).

### Statistical Analysis

The SPSS software (verion 20.0, IBM Corp, Armonk, NY, United States) and MedCalc software (version 15.2, Mariakerke, Belgium) were used to analyze the data. A statistically significant difference was considered as *p* value is less than 0.05. Figures were produced using GraphPad Prism (version 8.0, GraphPad Software, San Diego, CA, United States). The classification data were expressed as frequencies; Continuous variables were expressed as means and standard deviations. The diagnostic sensitivity, specificity, accuracy, positive predictive values (PPVs), negative predictive values (NPVs) of the CAD software were calculated by comparing the pathological results. McNemar’s test was used to compare the diagnostic sensitivity, specificity and accuracy of the CAD system and the senior and junior radiologists. The diagnostic performance of the radiologist assisted by the CAD system was defined as positive when the criteria meet one of the two categories: the radiologist and the CAD system. The diagnosis performance concerning nodule size was compared using the chi-squared (χ^2^) test with Bonferroni adjustment, which result in a final *P*-value of 0.0167, based on three independent tests, considered statistically significant. The areas under the receiver operating characteristic (ROC) curve (AUC) were also analyzed to compare the diagnostic performance of different groups, and optimal cut-off value for CAD was defined by the Youden index J.

## Results

### Patients Data

A total of 303 patients (Mage = 46.4 years; range 23–80 years) with 365 thyroid nodules were included in this study (Table 1). There were 186 cases (51.0%) that were malignant, including 168 cases of papillary carcinoma, 11 cases of follicular carcinoma, and 7 cases of medullary carcinoma. There were 179 cases (49.0%) that were benign, including 127 cases of follicular adenoma, and 52 cases of nodular goiters.

**TABLE 1:** Characteristics of study subjects.

Parameter	Value
Mean age (years)	46.4 ± 14.6
**Patient gender, *n* (%)**	
Male	59 (19.5%)
Female	244 (80.5%)
**No. of nodules, *n* (%)**	
Benign nodules	179 (49%)
Malignant nodules	186 (51%)
**Nodule sizes (mm)**	
Total nodules	18.33 ± 13.5
Benign nodules	25.58 ± 13.7
Malignant nodules	11.36 ± 8.8

### CAD Score Cut-Off Value for Predicting Malignant Thyroid Nodules

**FIGURE 2:**
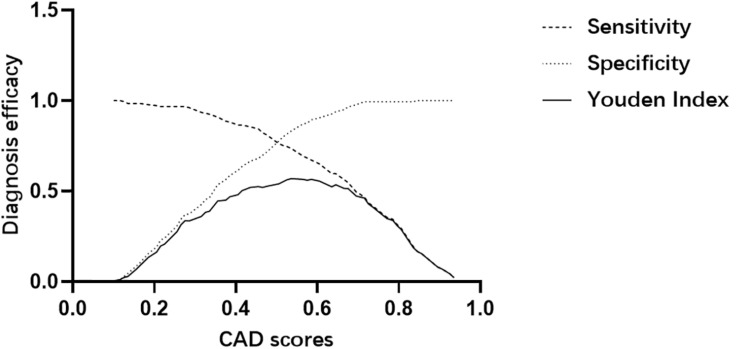
Determination of the positive threshold for the CAD system score through the Youden index.

We determined the positive threshold of CAD scores using the Youden Index (= sensitivity + specificity-1) curve, which could suggest the best cut-off value by fitting optimal sensitivity and specificity (11). The maximum Youden Index pointed to 0.555 for the CAD system score with a sensitivity of 71.5% and a specificity of 86.0% (Figure 2).

### Diagnostic Performance of the CAD System, Radiologists in the Different Groups and CAD-Assisted Radiologists

**FIGURE 3:**
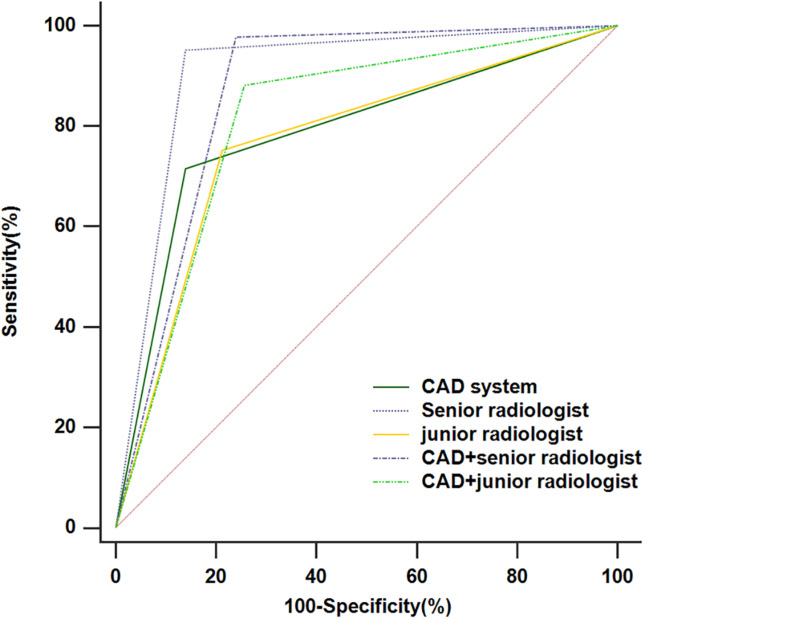
The receiver operating characteristic (ROC) curves for the performance of the computer-aided diagnosis (CAD) system, the senior radiologist, the junior radiologist, and CAD-assisted radiologists.

The diagnostic performances of the CAD system, radiologists in the different groups, and CAD-assisted radiologists for detecting thyroid cancer were summarized in Table 2 and Figure 3. The CAD system exhibited no statistically significant difference in terms of specificity compared with the senior radiologist (86.0% vs. 86.0%, *p* > 0.99), while the sensitivity and accuracy were markedly lower in the CAD system than those in the senior radiologist (71.5% vs.95.2%, *p* < 0.001; 78.6% vs.90.7%, *p* < 0.001, respectively). When compared with the junior radiologist, the CAD system resulted in increased specificity and similar sensitivity and accuracy in the classification of thyroid cancer (86.0% vs.78.8%, *p* = 0.024; 71.5% vs.75.3%, *p* = 0.419; 78.6% vs.77.0%, *p* = 0.552, respectively). When the CAD system was used to assist the senior and junior radiologists, the diagnostic sensitivity improved (97.8% vs. 95.2%, *p* = 0.063; 88.2% vs. 75.3%, *p* < 0.001, respectively), while the specificity declined (76.0% vs. 86.0%, *p* < 0.001; 79.9% vs. 84.4%, *p* = 0.008, respectively). A ROC analysis comparing the diagnostic values of the CAD system, radiologists, and CAD-assisted radiologists is illustrated in Figure 3 and Table 2. The AUCs were 0.788 (0.742, 0.829) for the CAD system, 0.906 (0.871, 0.934) for the senior radiologist, 0.869 (0.830, 0.902) for the CAD-assisted senior radiologist, 0.770 (0.724, 0.812) for the junior radiologist, and 0.812 (0.768, 0.851) for the CAD-assisted junior radiologist.

**TABLE 2:** Diagnostic performance of CAD System, radiologists and CAD-assisted radiologists.

Diagnostic measures (%)	Sensitivity	Specificity	PPV	NPV	Accuracy	Area under the ROC curve
CAD system	71.5 (133/186)	86.0 (154/179)	84.2 (133/158)	74.4 (154/207)	78.6 (287/365)	0.788
Senior radiologist	95.2 (177/186)	86.0 (154/179)	87.6 (177/202)	94.5 (154/163)	90.7 (331/365)	0.906
CAD + senior radiologist	97.8 (182/186)	76.0 (136/179)	80.9 (182/225)	97.1 (136/140)	87.1 (318/365)	0.869
Junior radiologist	75.3 (140/186)	78.8 (141/179)	78.7 (140/178)	75.4 (141/187)	77.0 (281/365)	0.770
CAD + Junior radiologist	88.2 (164/186)	74.3 (133/179)	78.1 (164/210)	85.8 (133/155)	81.4 (297/365)	0.812
*P*-value*	<0.001	>0.99			<0.001	<0.001
*P*-value**	0.063	<0.001			0.011	0.031
*P*-value^‡^	0.419	0.024			0.586	0.552
*P*-value^†^	<0.001	0.008			0.121	0.022
*P*-value^†^	0.015	0.003			<0.001	<0.001

*P*-value* is that of the CAD system vs. the senior radiologist; *P*-value** is that of the CAD-assisted senior radiologist vs. the senior radiologist; *P*-value^‡^ is that of the CAD system vs. the junior radiologist; *P*-value^†^ is that of the CAD-assisted junior radiologist vs. the junior radiologist; *P*-value^†^ is that of the CAD-assisted junior radiologist vs. the senior radiologist; NPV, negative predictive value; PPV, positive predictive value.

### Comparison of the Diagnostic Performance of the CAD System for the Diagnosis of Thyroid Nodules of Different Sizes

The sensitivity, specificity, NPV, PPV, and accuracy of the CAD system for diagnosing thyroid nodules of different sizes were summarized in Table 3. The sensitivity, specificity, and accuracy of the CAD system for the diagnosis of small thyroid nodules diagnosis did not differ from those of medium-sized thyroid nodules after applying Bonferroni correction (*P* = 041; *P* = 0.025; *P* = 0.818, respectively). The sensitivity of the CAD system for the diagnosis of large thyroid nodules was significantly less than for small thyroid nodules (*P* < 0.0167), whereas the specificity and the accuracy of the CAD system in the diagnosis of large thyroid nodules were significantly higher than that of small thyroid nodules (*P* < 0.001; *P* < 0.001, respectively). The sensitivity and specificity of the CAD system for the diagnosis of large thyroid nodules diagnosis did not differ from those of medium-sized thyroid nodules (*P* = 0.486; *P* = 0.062, respectively).

**TABLE 3:** Comparison of the diagnostic performance of the CAD system in differentiating the thyroid nodules of different sizes.

	Sensitivity (%)	Specificity (%)	Accuracy (%)
*d* ≤ 15 mm	76.3 (116/152)	65.5 (36/55)	73.4 (152/207)
15 < *d* ≤ 25 mm	55.0 (11/20)	87.5 (28/32)	75 (39/52)
*d* > 25 mm	42.9 (6/14)	97.8 (90/92)	90.5 (96/106)
*P*-value			
*d* ≤ 15 mm vs. 15 < *d* ≤ 25 mm	0.041	0.025	0.818
*d* ≤ 15 mm vs. *d* > 25 mm	0.016*	<0.001*	<0.001*
15 < *d* ≤ 25 mm vs. *d* > 25 mm	0.486	0.062	0.009*

*P values considered significant after applying Bonferroni correction.

## Discussion

Ultrasonography is playing a crucial role in the greatly increasing detection rate of thyroid nodules (12, 13). However, the usefulness of ultrasound may be limited for the diagnostic performance of it is various from person to person, which depends on the experience of a radiologist to a large extent (14). The original CAD system was used to diagnose the breast tumor in the 1960s (15). The CAD system which based on artificial intelligence has been developed to assist radiologists in analyzing images, shortening the time cost of the diagnostic process, and reducing interobserver variability.

In this study, a clinical assessment was performed to evaluate the value of an ultrasound CAD system in the ultrasound diagnosis of thyroid cancer. This retrospective study showed that the CAD system generally performed comparably to qualitative assessments by the senior radiologist in terms of specificity, but had a lower sensitivity and accuracy. In addition, the specificity of the CAD system was greatly higher than that of the junior radiologist and the CAD system demonstrated similar sensitivity and accuracy to the junior radiologist.

Since the diagnostic performance for thyroid lesions of the CAD system initially reported (16), several studies have already revealed that CAD approaches improved the diagnostic manifestations of thyroid ultrasound (8, 9, 17–19). More recently, Chung et al. compared the diagnostic performance of a real-time CAD system with that of a 7-year experienced radiologist, CAD system had comparable sensitivity but lower specificity than the experienced radiologist (20). However, Gitto et al. reported that the CAD system had a significantly lower sensitivity than the experienced radiologist and there was no statistical difference in specificity (21).

The added value of the CAD system was also evaluated in this study. With the assistant of the CAD system, the junior radiologist showed a significant increase in sensitivity from 75.3 to 88.2%. Also, the AUC was greatly improved from 0.770 to 0.812 (*P* = 0.022). The improved sensitivity, NPV, and AUC indicated that the CAD system might function as a supplementary opinion to avoid the missed diagnosis, especially for less-experienced radiologists. As was shown in the study, the CAD system had a comparable specificity to that of a senior radiologist, which implied that the CAD system could play a constructive role in avoiding overdiagnosis and help to reduce unnecessary biopsies for the thyroid nodule diagnosis.

In this study, we further analyzed whether the efficiency of diagnosis of the CAD systems were affected by nodule sizes. It was shown that the diagnostic performance of the CAD system was not consistent in each group depending on the size of the lesion. The sensitivities of the CAD system in identifying small were significantly higher than those of large nodules. These results may be attributed to that large thyroid nodules tend to occupy most of the thyroid gland in the US image, which makes it hard for the CAD system to distinguish between the nodules and the normal thyroid gland. This should be considered when the CAD system is used in clinical practice.

The study contributes to several clinical implications. First, the CAD system in this study can automatically recognize and analyze the thyroid nodules of US images, which demonstrates an opportunity for the combination between clinician and machine in future clinical practice. Second, the CAD system exhibited no statistically significant difference in terms of specificity compared with the senior radiologist, although the sensitivity was lower. This finding implied that the CAD system could cut down unnecessary biopsies and also help to lighten the load of physicians. Besides, the use of the CAD system significantly improved the diagnostic sensitivity and AUC of the junior radiologist, which suggested the possibility that it could serve as a second opinion for less experienced radiologists to minimize missed diagnosis. Lastly, the diagnostic efficiency of the CAD system for thyroid nodules of different sizes was evaluated, which was able to reflect the clinical value of the CAD system further.

This study also has some limitations. First of all, the sample capacity was relatively small and selection bias was inevitable due to the retrospective study nature. Second, the diagnostic criteria for the CAD system-assisted radiologist diagnosis are artificially defined. The actual help of the CAD system for the radiologists in clinical needs to be substantiated in the future. Further, although this study enrolled five pathological types of thyroid nodules, most of the malignant nodules were PTCs. However, the follicular thyroid carcinoma appears with different sonographic characteristics from PTC and tend to show more benign US features (22, 23), which make it difficult for CAD systems to distinguish FTC from thyroid nodules. Large-scale multicenter studies are needed to overcome these drawbacks and generalize the findings.

In conclusion, the CAD system assessed in this study shows comparable specificity to that of the senior radiologist and helps to improve the diagnostic sensitivity and AUC of the junior radiologist significantly. The nodule size of thyroid nodules are potential influencers of CAD diagnostic performance. Further efforts are required to improve its diagnostic performance and future researches are necessary to evaluate the clinical role of CAD in thyroid nodule diagnosis.

## Data Availability Statement

The raw data supporting the conclusions of this article will be made available by the authors, without undue reservation.

## Ethics Statement

The study was reviewed and approved by Ethics Committee of Shanghai Sixth People’s Hospital. All procedures performed in the study involving human participants were in accordance with the ethical standards of the institutional research committee and the 1964 Helsinki declaration and its later amendments or comparable ethical standards. Formal consent is not required for this type of study.

## Author Contributions

YZ and QW were major contributors in writing the manuscript. YZ, QW, and YW conceived and designed the experiments. YZ did the literature research. QW analyzed the data. YC provided basic information on all cases. All authors contributed to the article and approved the submitted version.

## Conflict of Interest

The authors declare that the research was conducted in the absence of any commercial or financial relationships that could be construed as a potential conflict of interest.
